# Limited phylogeographic and genetic connectivity in *Acacia* species of low stature in an arid landscape

**DOI:** 10.1002/ece3.9052

**Published:** 2022-07-06

**Authors:** Melissa A. Millar, Rachel M. Binks, Sarah‐Louise Tapper, Bronwyn M. Macdonald, Shelley L. McArthur, Margaret Hankinson, David J. Coates, Stephen van Leeuwen, Margaret Byrne

**Affiliations:** ^1^ Department of Biodiversity, Conservation and Attractions Biodiversity and Conservation Science Bentley Western Australia Australia; ^2^ School of Molecular and Life Sciences Curtin University Perth Western Australia Australia

**Keywords:** *Acacia*, arid zone, genetic connectivity, genetic diversity, Pilbara, stature

## Abstract

Widespread plant species are expected to maintain genetic diversity and gene flow via pollen and seed dispersal. Stature is a key life history trait that affects seed and potentially pollen dispersal, with limited stature associated with limited dispersal and greater genetic differentiation. We sampled Hill’s tabletop wattle (*Acacia hilliana*) and curry wattle (*Acacia spondylophylla*), two co‐distributed, widespread, *Acacia* shrubs of low stature, across the arid Pilbara region of north‐western Australia. Using chloroplast sequence and nuclear microsatellite data we evaluated patterns of population genetic and phylogeographic diversity and structure, demographic signals, ratios of pollen to seed dispersal, evidence for historical refugia, and association between elevation and diversity. Results showed strong phylogeographic (chloroplast, *G*_ST_ = 0.831 and 0.898 for *A. hilliana* and *A. spondylophylla*, respectively) and contemporary (nuclear, *F*_ST_ = 0.260 and 0.349 for *A. hilliana* and *A. spondylophylla*, respectively) genetic structure in both species. This indicates limited genetic connectivity via seed and pollen dispersal associated with *Acacia* species of small stature compared to taller tree and shrub acacias across the Pilbara bioregion. This effect of stature on genetic structure is superimposed on moderate levels of genetic diversity that were expected based on widespread ranges (haplotype diversity *h* = 25 and 12; nuclear diversity *H*e = 0.60 and 0.47 for *A. hilliana* and *A. spondylophylla*, respectively). Contemporary genetic structure was congruent at the greater landscape scale, especially in terms of strong genetic differentiation among geographically disjunct populations in less elevated areas. Measures of diversity and connectivity were associated with traits of greater geographic population proximity, population density, population size, and greater individual longevity, and some evidence for range expansion in *A. hilliana*. Results illustrate that low stature is associated with limited dispersal and greater patterns of genetic differentiation for congenerics in a common landscape and highlight the complex influence of taxon‐specific life history and ecological traits to seed and pollen dispersal.

## Graphical abstract

We assessed phylogeographic and population genetic diversity and structure in two Acacia shrubs of low stature with widespread ranges and co‐distributions across the arid Pilbara region of north‐western Australia: Hill’s tabletop wattle (*Acacia hilliana*) and curry wattle (*Acacia spondylophylla*). Both species showed strong phylogeographic and contemporary genetic structure indicating reduced genetic connectivity when compared to taller shrub and tree Acacias in the Pilbara but maintained levels of genetic diversity comparable to that of other widespread Acacia

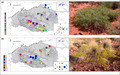

## 1 INTRODUCTION

Understanding drivers of species genetic diversity, differentiation and demography is of central interest to the fields of biogeography, phylogeography, population and conservation genetics, and ecology (Avise, 2000). Species with sympatric regional biogeographic distributions are expected to have experienced the same historical geographic, geological, and/or climatological phenomena across landscapes (Arbogast & Kenagy, 2001). As a result, comparative phylogeographic analyses of broadly co‐distributed species can assist in elucidating the impact of regional geological and other landscape features, as well as historical macroevolutionary climatic and environmental events, on patterns of genetic diversity, differentiation, and demography at the landscape scale (Allendorf et al., 2013; Arbogast & Kenagy, 2001; Avise, 2000). Identifying common patterns in areas of haplotype diversity and divergence can lead to the identification of landscape features that may have provided potential historical refugia, or barriers to dispersal, as well as patterns of population persistence, contraction, and/or expansion (Hickerson et al., 2010; Keppel et al., 2012).

Identifying macroevolutionary patterns of biogeographic concordance among co‐distributed taxa should also be balanced by biologically informed approaches that determine the contribution of taxon specific life history and ecological traits on levels and structuring of genetic diversity on more microevolutionary temporal and spatial scales (Papadopoulou & Knowles, 2016; Sullivan et al., 2019). For example, plant species are influenced to varying degrees by a range of life‐history traits that drive fine‐scale processes of seed and pollen dispersal, as well as by ecological traits (Broadhurst et al., 2017; Hamrick & Godt, 1996). Key life history traits that affect seed and pollen dispersal include mating systems (Duminil et al., 2007), the mechanisms of dispersal (Duminil et al., 2007; Thomson et al., 2010), and stature (Petit & Hampe, 2006; Thomson et al., 2010). Meta‐analyses have shown these key traits, as well as geographic and demographic attributes of range size, degree of population geographic disjunction, localized abundance or density, population size, and individual longevity and fecundity, have the greatest effects on genetic diversity and differentiation (Broadhurst et al., 2017; Gitzendanner & Soltis, 2000; Hamrick, 1979; Hamrick & Godt, 1996; Hamrick et al., 1992; Loveless & Hamrick, 1984; Nybom, 2004; Nybom & Bartich, 2000). The use of comparative phylogeography in deducing how differing life history and ecological traits contribute to macro and microevolutionary patterns of genetic diversity, differentiation, and demography of specific plant taxa is not common (although see García‐Verdugo et al., 2019; Massatti & Knowles, 2014; Paz et al., 2015). Comparative approaches that assess concordance in contemporary patterns of genetic diversity driven by trait based microevolutionary processes, as well as historical abiotic macroevolutionary processes, offer fundamental insights into the taxa themselves and allow for refinement of predictions on how taxon‐specific traits affect diversity, differentiation, and demography (Papadopoulou & Knowles, 2016).

Historical evolution in a common landscape is typically expected to result in concordant biogeographic patterns (Arbogast & Kenagy, 2001; Hewitt, 2004). However, the influence of intrinsic taxon‐specific life history and ecological traits on phylogeographic patterns, as well as contemporary population genetic diversity and differentiation, is often underestimated (Papadopoulou & Knowles, 2016). Biogeographic studies are also underrepresented in many species‐rich environments, particularly in the Southern Hemisphere (Beheragaray et al., 2008; Hickerson et al., 2010), in Australia (Beheragaray et al., 2008), and in the world’s arid zones (Byrne et al., 2008; Lexer et al., 2013; Robertson & Chan, 2011). The Pilbara bioregion of north‐western Australia provides an opportunity to investigate comparative genetic patterns in a species‐rich, topographically complex, arid environment in the Southern Hemisphere. Previous studies of three *Acacia* present in the Pilbara have shown evidence for congruent patterns of historical population isolation and persistence at the broad regional scale (Levy et al., 2016; Nistelberger et al., 2020). However, patterns of phylogeographic and contemporary population genetic diversity and differentiation have also been shown to vary with differences in taxon‐specific life history and ecological traits known to impact pollen and seed dispersal, including differences in species geographic range size, and growth form or stature. For example, species with widespread ranges, *A. ancistrocarpa* and *A. pruinocarpa*, show greater levels of chloroplast haplotype diversity, haplotype network complexity, phylogeographic structure, and contemporary genetic diversity and connectivity (Levy et al., 2016; Nistelberger et al., 2020), compared to the range restricted *A. atkinsiana* (Levy et al., 2016). Also, the tallest of the three species, *A. pruinocarpa* (to 12 m), has increased haplotype diversity, a more complex haplotype network, and some evidence for population expansion (Nistelberger et al., 2020) in comparison to the smaller shrub species (both to 4 m) that show more limited haplotype network diversity, and evidence for population persistence (Levy et al., 2016).

**FIGURE 1:**
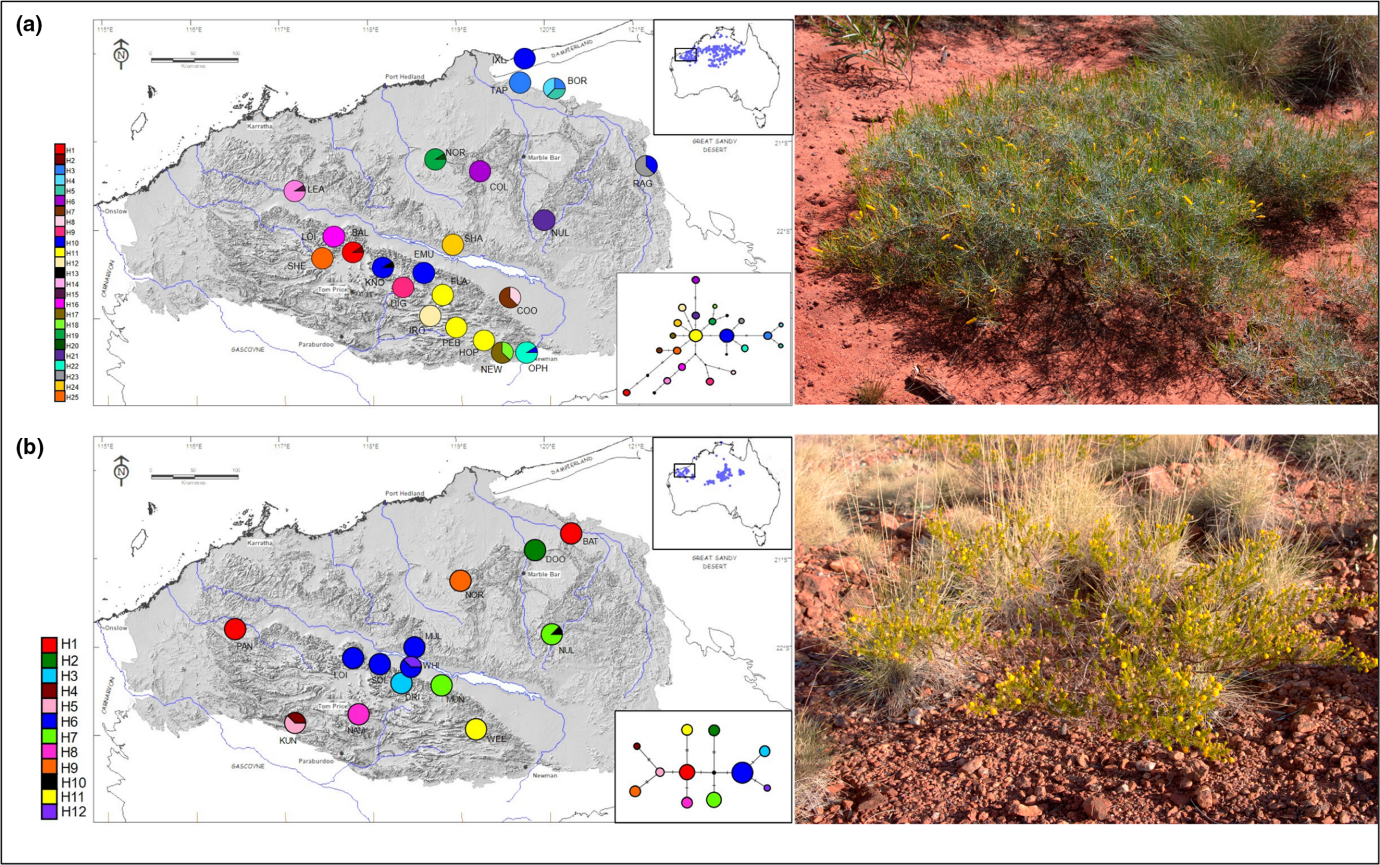
Geographic distribution of sampled populations, chloroplast DNA haplotypes, and evolutionary relationships amongst chloroplast DNA haplotypes of (a) *Acacia hilliana* and (b) *Acacia spondylophylla* in the Pilbara bioregion. Populations are represented by pie charts. Segment size within each pie‐chart corresponds to the proportion of individuals with that haplotype. Population codes correspond to those in Table 1. Greyscale indicates elevation. Top inset shows species known geographic distribution as blue circles. Pilbara bioregion indicated. Bottom inset Median‐Joining networks show the evolutionary relationships between cpDNA haplotypes in sampled populations. Haplotypes are represented by colored circles. Color of circle corresponds to haplotypes in the geographic map. Circle size is proportional to the number of individuals with that haplotype and branch lengths are proportional to the number of mutations with lengths greater than one indicated by cross bars. Small black circles indicate unsampled hypothetical haplotypes. Panels to the right show the prostrate nature of representative individuals

Limiting comparisons to congeneric species that share key life history traits that primarily affect dispersal allows for refined hypotheses regarding the contributions of life history and ecological traits to diversity, differentiation, and demography. Here, we test hypotheses based on intrinsic taxon‐specific life history and ecological traits to improve understanding of macro and microevolutionary drivers of genetic diversity and differentiation for *Acacia* species in the Pilbara. We use both chloroplast (cpDNA) sequencing data and nuclear microsatellite (nDNA) data to assess intraspecific patterns of phylogeographic and population genetic diversity and structure in Hill’s tabletop wattle (*Acacia hilliana* Maiden, Figure 1a) and curry wattle (*Acacia spondylophylla* F. Muell. Figure 1b). *Acacia hilliana* and *A*. *spondylophylla* are regionally common, with widespread, broadly co‐distributed geographic ranges within the Pilbara (Figure 1). Both species have widespread ranges extending outside the Pilbara region suggesting that haplotype and nuclear diversity will be comparable to similarly widespread *Acacia* species (Levy et al., 2016; Nistelberger et al., 2020). However, unlike the *Acacia* species studied so far, which are large shrubs or trees, *A. hilliana* and *A. spondylophylla* are both small, flat‐topped shrubs less than 1.5 m tall. A global review has shown that low stature is associated with significant limitations to seed dispersal distance for species with the same seed dispersal syndrome (Thomson et al., 2011). Given that *Acacia* in the arid Pilbara may be expected to have similar key life history traits that primarily affect pollen and seed dispersal, a predominantly outcrossed mating system, pollination by generalist insects and seed dispersal primarily by gravity and ants, low stature may be expected to result in greater limitation to seed dispersal and greater phylogeographic structure for the study species. The two study species differ in other life history and ecological traits including the degree of population geographic disjunction, localized population density, population size, and individual longevity and fecundity (detailed in the Materials and Methods), that may also result in observable differences in contemporary patterns of genetic diversity and differentiation between the species.

We use a standardized comparative approach at macro and microevolutionary spatial and temporal scales to gain insight into how levels and patterns of genetic diversity are influenced by taxon‐specific life history and ecological traits. Our assessment of two widespread *Acacia* shrubs of small stature is placed in the context of highly comparable studies, that used a similar suite of chloroplast sequencing data and nuclear microsatellite data and the same sampling approach and sampling numbers, on several *Acacia* with variable species ranges across the Pilbara and that are either trees or large shrubs. We make the following predictions for *A. hilliana* and *A. spondylophylla*; (i) they will exhibit levels of haplotype and nuclear diversity and contemporary genetic connectivity comparable to those of other widespread *Acacia* present in the Pilbara; (ii) given that low growing habit likely leads to more restricted seed dispersal, they will exhibit stronger phylogeographic structure compared to that of tall shrub and tree *Acacia* present in the Pilbara. Patterns of contemporary genetic structure and diversity are likely to be largely congruent for *A. hilliana* and *A. spondylophylla* as they have experienced the same historical geographic, geological, and climatological phenomena at the regional scale, although a greater degree of population disjunction, lower localized population density, smaller populations, and shorter individual longevity and lower fecundity in *A. spondylophylla* leads to another prediction of (iii) decreased genetic diversity within populations and increased genetic variation among populations of *A. spondylophylla*. We also assessed phylogeographic concordance for *A. hilliana* and *A. spondylophylla* in terms of the major landscape features of the Hamersley and Chichester Ranges, that may act as potential historical refugia, and the impact of population elevation on measures of contemporary diversity.

## 2 MATERIALS AND METHODS

### 2.1 Study region

The Pilbara bioregion of Western Australia is a highly structured landscape, recognized as a biogeographic transition zone between the tropics to the north, the sandy deserts to the east and the arid rangelands to the south (Cracraft, 1991; Pepper et al., 2013). The region supports a wide range of habitats with a climate dominated by high temperatures and limited annual rainfall, although localized flooding can result from thunderstorms that often follow tropical summer cyclones (Leighton, 2004). Key landscape features are the Hamersley and Chichester Ranges that run east to west some 460 km, and the Fortescue River Valley that bisects them (McKenzie et al., 2009; Pepper et al., 2013). The uplifted iron rich sedimentary Hamersley Range (up to 1249 m) comprises a series of mountainous ranges, ridges, eroded gorges, and hills with an elevated central plateau. Vegetation of the Hamersley Range is largely comprised of scattered emergent eucalypts, *Acacia* trees and shrubs over spinifex (*Triodia* spp.) hummock grasslands. To the north, the basaltic, granitic/greenstone Chichester Range (up to 367 m) comprises an escarpment and tableland with jagged peaks, gorges, rolling hills, and tree‐lined water courses with spinifex, hummock grasslands and *Eucalyptus leucophloia* steppe. The region is recognized as a significant center of species endemism across the flora and fauna biota of the Australian continent (Burbidge et al., 2010; Cracraft, 1991; Doughty et al., 2011; Durrant et al., 2010; Eberhard et al., 2005; Gibson & McKenzie, 2009; Guthrie et al., 2010; Ladiges et al., 2006; McKenzie & Bullen, 2009; Unmack, 2001). The Pilbara is also an important center of diversity for the *Acacia* genus (Maslin et al., 2010), whose species are common in Australian arid and semi‐arid zone plant communities (Beadle, 1981).

### 2.2 Study species

Hill’s tabletop wattle (*A. hilliana*) is a semi prostrate sub‐shrub reaching 0.3–1 m in the Pilbara. Plants have bright yellow, erect, cylindrical flower spikes to 45 mm from April to November (Maslin et al., 2010). Brown seeds up to 5.5 mm form in erect, woody, resinous pods that produce a light but distinct citrus odor (Maslin et al., 2010). Each seed has a large eliasome and seed is shed from pods following dehiscence. Individuals are slow growing, expected to live 50+ years, and are highly fecund (seed production). The species has a widespread but scattered distribution from the Pilbara through central and northern Australia to north‐western Queensland (Figure 1a). Plants occur on skeletal red loamy sand or sand over laterite, quartzite, or ironstone, on sand dunes and on spinifex plains in deep sand on the lowlands, but also on rocky elevated sites on the ranges and plateaus of the Pilbara (Maslin et al., 2010). Populations are generally large, covering many hectares, and are continuous and locally dense on lowland rolling hills and upland plains of the ranges, although populations off the ranges are less continuous with some disjunctions (S van Leeuwen, personal observation).

Curry wattle (*Acacia spondylophylla*) grows slightly larger but is still a small, spindly shrub typically 1–1.5 m. Plants have resinous leaves, stems, and pods that produce a curry‐like odor, with larger, bright yellow, round inflorescences appearing from May to August (Maslin et al., 2010). Slightly smaller black seeds, 3.5–4 mm long, with a small eliasome form within flat pods and are easily shed from pods (Maslin et al., 2010). Individuals are fast growing, living 5–15 years, and fecundity is limited with relatively few seeds being produced. The species has a scattered distribution across the Pilbara and central Australia (Figure 1b), with typically open and sparse populations that are more disjunct, less dense, and smaller than those of *A. hilliana* (S van Leeuwen, personal observation). Plants grow in shallow, sandy or rocky soil on ironstone hills and in gullies, as well as along creeks and colluvial flats on spinifex plains (Maslin et al., 2010). Populations are patchily distributed or disjunct and occur with higher density on the Hamersley Range, although elevated populations are still more scattered, less dense, and smaller than those of *A. hilliana* (S van Leeuwen, personal observation).

These species are expected to have the same key life history traits as other Pilbara *Acacia* studied so far. Although there are exceptions, most *Acacia* share life history traits that have primary effects on dispersal of both seed and pollen. These include pollen dispersal by generalist insect pollinators (Bernhardt, 1989; Stone et al., 2003), self‐incompatibility, or at least partial self‐incompatibility that may be prezygotic or postzygotic (Kenrick & Knox, 1989; Morgan et al., 2002; New, 1984), and seed dispersed primarily by gravity and secondarily by myrmecochory (Berg, 1975; Davidson & Morton, 1984).

### 2.3 Sampling and DNA extraction

Distributional information on each species across the Pilbara bioregion was determined from specimens lodged at the Western Australian Herbarium through the FloraBase website (http://florabase.dpaw.wa.gov.au). Sampling sites (herein populations) were selected to cover the distributional range of each species across the Pilbara. Collections of phyllode material were made from 24 plants in each of 22 populations of *A. hilliana* and 14 populations of *A. spondylophylla* (Table 1). Relevant fieldwork permission was obtained from landowners and samples collected under license numbers SW014833 and SW015514 issued by the Department of Biodiversity, Conservation and Attractions. Individuals of each species were readily identified in the field, except for individuals in one population later identified as hybrids (see below). Voucher specimens for both species were collected at each sampled population and lodged with the Western Australian Herbarium. Phyllode material was freeze‐dried. DNA of *A. hilliana* was extracted using a modified CTAB method (Doyle & Doyle, 1990), adding 1% PVP40 (polyvinylpyrrolidone) and 0.1M sodium sulfite to the extraction buffer. DNA of *A. spondylophylla* was extracted using the Stratec Invisorb® DNA Plant HTS 96 Kit/C (STRATEC Molecular GmbH).

**TABLE 1:** Geographical parameters and estimates of genetic diversity in two chloroplast regions and 16 nuclear microsatellite loci calculated for sampled populations of *Acacia hilliana* and *Acacia spondylophylla* in the Pilbara bioregion. Latitude, Longitude, and elevation (m) are given. Location: on the Hamersley Range (H), on the Chichester Range (C), off the ranges (O). Chloroplast diversity: the number of haplotypes present (*H*), the number of private haplotypes (*H*p). The haplotype number of haplotypes present in each population is also given (Hap#). Microsatellite diversity: allelic richness (*N*_R_), the number of private alleles (*N*_AP_), observed (*H*o) and expected (*H*e) heterozygosity, the proportion of polymorphic loci (%*P*), and the Fixation index (*F*_IS_). Standard errors in parentheses

*Acacia hilliana*						Chloroplast diversity			Microsatellite diversity					
Population	Code	Latitude	Longitude	Elevation	Location	*H*	*H*p	Hap#	*N*_R_	*N*_*AP*_	*Ho*	*He*	%*P*	*F*_IS_
Balbina	BAL	22°13′ 52.9″S	117°53′ 03.9″E	639	H	2	2	1, 2	5.93 (0.75)	2	0.51 (0.06)	0.64 (0.06)	100.00	0.18 (0.05)
Boreline	BOR	20°27′ 14.2″S	120°10′ 29.3″E	178	O	3	2	3, 4, 5	4.71 (0.74)	—	0.46 (0.08)	0.51 (0.07)	87.50	0.11 (0.08)
Coolyia	COL	21°23′ 24.1″S	119°33′ 38.5″E	300	O	1	1	6	5.71 (0.77)	2	0.50 (0.07)	0.58 (0.07)	93.75	0.10 (0.06)
Coondiner	COO	22°42′ 53.2″S	119°40′51.8″E	420	H	2	2	7, 8	5.51 (0.68)	1	0.57 (0.06)	0.63 (0.05)	100.00	0.10 (0.04)
Digman	DIG	22°33′ 56.6″S	118°29′ 27.6″E	747	H	1	1	9	6.63 (0.72)	1	0.71 (0.04)	0.71 (0.04)	100.00	−0.02 (0.04)
East Munjina	EMU	22°29′ 22.5″S	118°44′ 10.3″E	687	H	1	—	10	6.63 (0.68)	5	0.64 (0.05)	0.71 (0.05)	100.00	0.11 (0.04)
Flat Rocks	FLA	22°43′ 44.6″S	118°57′00.4″E	630	H	1	—	11	5.48 (0.68)	—	0.57 (0.07)	0.61 (0.06)	93.75	0.06 (0.06)
Hope Downs	HOP	23°13′ 57.2″S	119°30′ 01.0″E	735	H	1	—	11	5.52 (0.79)	—	0.59 (0.06)	0.64 (0.06)	93.75	0.06 (0.05)
Iron Ore	IRO	22°54′ 21.0″S	118°48′ 37.5″E	737	H	1	1	12	4.85 (0.65)	—	0.55 (0.07)	0.58 (0.05)	93.75	0.06 (0.06)
IXL Well	IXL	20°08′25.6″S	119°50′40.8″E	50	O	1	—	10	4.90 (0.63)	1	0.46 (0.06)	0.50 (0.07)	93.75	0.07 (0.04)
Knox	KNO	22°22′ 46.4″S	118°17′05.7″E	713	H	2	1	10, 13	7.05 (0.81)	1	0.61 (0.04)	0.75 (0.03)	100.00	0.19 (0.05)
Leal	LEA	21°34′ 29.6″S	117°19′ 16.4″E	363	O	2	2	14, 15	4.60 (0.64)	—	0.46 (0.05)	0.57 (0.06)	93.75	0.19 (0.07)
Lois	LOI	22°06′ 34.8″S	117°43′ 39.9″E	627	H	1	1	16	5.85 (0.88)	1	0.48 (0.04)	0.62 (0.04)	100.00	0.21 (0.04)
Newman	NEW	23°14′ 56.1″S	119°32′ 50.8″E	717	H	2	2	17, 18	5.95 (0.62)	1	0.58 (0.05)	0.70 (0.04)	100.00	0.16 (0.04)
North Star	NOR	21°14′ 39.5″S	118°53′ 50.5″E	206	O	2	2	19, 20	5.40 (0.71)	3	0.56 (0.05)	0.65 (0.05)	93.75	0.12 (0.05)
Nullagine	NUL	21°53′ 00.5″S	120°05′ 39.9″E	387	O	1	1	21	4.75 (0.65)	2	0.56 (0.06)	0.54 (0.05)	93.75	−0.07 (0.04)
Ophthalmia	OPH	23°18′ 58.3″S	119°51′44.4″E	521	H	2	1	22, 10	5.01 (0.75)	—	0.50 (0.06)	0.54 (0.07)	87.50	0.03 (0.05)
Pebble	PEB	23°01′ 44.3″S	119°04′39.2″E	647	H	1	—	11	4.74 (0.45)	—	0.51 (0.06)	0.57 (0.06)	100.00	0.09 (0.05)
Ragged	RAG	21°17′ 45.1″S	121°13′ 01.3″E	330	O	2	1	23, 10	6.81 (0.88)	8	0.57 (0.06)	0.64 (0.06)	100.00	0.12 (0.06)
Shaw	SHA	22°08′ 04.0″S	119°01′23.6″E	493	C	1	1	24	4.31 (0.73)	—	0.48 (0.07)	0.52 (0.07)	87.50	0.06 (0.04)
Sheila	SHE	22 15′ 01.8″S	117°38′23.8″E	642	H	1	1	25	5.02 (0.67)	1	0.48 (0.06)	0.55 (0.05)	100.00	0.10 (0.05)
Taplin	TAP	20°21′45.5″S	119°48′57.6″E	110	O	1	1	3	4.55 (0.62)	2	0.47 (0.08)	0.52 (0.07)	100.00	0.08 (0.06)
Total Mean						1.45	1.31		5.45 (0.17)*	2.21 (0.54)	0.54 (0.01)*	0.60 (0.02)*	96.02 (0.97)	0.100 (0.01)

Acacia spondylophylla						Chloroplast diversity			Microsatellite diversity					
Population	Code					H	Hp		*N*_R_	*N*_AP_	*Ho*	*He*	%P	*F*_IS_
Battler	BAT	20°43′25.2″S	120°14′064″E	116	O	1	—	1	3.10 (0.31)	3	0.51 (0.07)	0.47 (0.05)	93.75	−0.01 (0.10)
Doolena	DOO	20°55′11.8″S	119°48′45.8″E	143	O	1	1	2	3.25 (0.25)	1	0.40 (0.06)	0.43 (0.06)	93.75	0.02 (0.05)
Drillers	DRI	22°19′21.0″S	118°17′56.3″E	708	H	1	1	3	3.98 (0.52)	1	0.45 (0.07)	0.50 (0.07)	87.50	0.09 (0.04)
Kungarra	KUN	22°49′15.8″S	116°58′40.9″E	556	H	2	2	4, 5	3.98 (0.60)	2	0.44 (0.07)	0.47 (0.06)	93.75	0.08 (0.08)
Lois	LOI	22°06′34.8″S	117°43′39.9″E	628	H	1	—	6	2.41 (0.30)	1	0.35 (0.07)	0.33 (0.06)	75.00	−0.07 (0.05)
Mulga	MUL	22°02′20.4″S	118°24′03.6″E	441	C	1	—	6	3.88 (0.40)	3	0.41 (0.05)	0.47 (0.06)	100.00	0.12 (0.04)
Munjina	MUN	22°24′31.8″S	118°41′07.1″E	521	H	1	—	7	4.62 (0.44)	3	0.49 (0.05)	0.56 (0.06)	100.00	0.08 (0.05)
Nameless	NAM	22°43′11.1″S	117°44′59.9″E	915	H	1	1	8	3.63 (0.50)	1	0.35 (0.08)	0.40 (0.07)	87.50	0.12 (0.09)
Newman	NEW	23°14′14.3″S	119°31′22.1″E	733	H	—	—	—	4.14 (0.44)	12	0.40 (0.08)	0.54 (0.06)	93.75	0.24 (0.10)
North Star	NOR	21°15′22.8″S	118°55′54.0″E	234	O	1	1	9	4.74 (0.55)	3	0.50 (0.07)	0.57 (0.06)	100.00	0.12 (0.05)
Nullagine	NUL	21°53′00.5″S	120°05′39.9″E	392	O	2	1	10, 7	4.09 (0.53)	2	0.50 (0.07)	0.50 (0.06)	93.75	0.01 (0.06)
Pannawonica	PAN	21°46′29.5″S	116°17′01.6″E	172	O	1	—	1	3.21 (0.38)	2	0.34 (0.07)	0.42 (0.06)	81.25	0.23 (0.07)
Solomon	SOL	22°11′14.7″S	118°01′03.9″E	497	H	1	—	6	2.42 (0.30)	—	0.31 (0.07)	0.30 (0.07)	68.75	−0.03 (0.03)
Weeli Wolli	WEE	22°55′32.6″S	119°11′49.7″E	571	H	1	1	11	4.49 (0.46)	1	0.52 (0.07)	0.56 (0.06)	100.00	0.08 (0.06)
Wittenoom	WIT	22°14′54.3″S	118°21′33.2″E	470	O	2	1	12, 6	4.79 (0.53)	1	0.54 (0.06)	0.56 (0.05)	100.00	0.02 (0.05)
Total mean						1.21	1.13		3.78 (0.20)^a^	3.00 (0.76)	0.43 (0.02)^a^	0.47 (0.02)^a^	91.25 (2.50)	0.070 (0.02)

^a^ Values are significantly different among species.

### 2.4 Chloroplast sequencing

Non‐coding chloroplast regions known to show variability in Australian plant species (*rpl*32‐*trn*L, *trn*Q‐*rps*16, *trn*V‐*ndh*C, *ndh*F‐*rpl*32, *ndh*F‐*trn*L, *psb*D‐*trn*T and *trn*S‐*trn*G, D4‐loop intron, *ndh*A, *Pet*B*‐rp1*16, and the *psb*A‐*trn*H region widely used for barcoding) were tested in each species with DNA amplification and analysis following Byrne and Hankinson (2012). Amplicons were sequenced using forward and reverse primers by the Australian Genome Research Limited Facility (AGRF, Perth, Australia, https://agrf.org.au). Regions were selected for further analysis based on sequence quality and nucleotide diversity. Eight individuals randomly selected from each population were sequenced at each region using *ndh*A*, psb*A‐*trn*H, *rpl*32‐*trn*L and *trn*V‐*ndh*C for *A*. *hilliana,* and *Pet*D, *rpl*32‐*trn*L and *trn*V‐*ndh*C for *A*. *spondylophylla*. Sequence reactions were carried out following Byrne and Hankinson (2012) with thermocycling conditions according to Shaw et al. (2007), with a Serapure method to purify PCR products (Faircloth & Glenn, 2014; Rohland & Reich, 2012). Sequence data were concatenated, aligned using Clustal W v2.0.11 (Thompson et al., 1994), and corrected manually where necessary using BioEdit (Hall, 1999). Variable sites were scored as a single mutation and indels coded as single binary states regardless of length. Indels arising from mononucleotide repeats were removed. These sequence data are available at the GenBank (www.ncbi.nlm.nih.gov/genbank) database under accession numbers MW138725‐MW138756 and MW696776‐MW696791.

### 2.5 Nuclear microsatellite library construction, primer assessment, and locus assessment

Species specific nuclear microsatellite markers were developed for each species through partial genome sequencing on a 454 platform at the AGRF, using the methods of Gardner et al. (2011). The resulting microsatellite libraries were sorted for functionality and the best 40 primers tested for polymorphism in a subset of individuals from multiple populations. Primer pairs were optimized for 16 reliable microsatellite loci for each species (Table S1). Microsatellite amplification was performed in 7.25 μl multiplexed reactions containing 3.5 μl QIAGEN Multiplex PCR Master Mix (Qiagen), 0.75 μl primer mix, 2 μl sterile distilled water and 1 μl (10 ng) of template DNA. Cycling conditions were the same for all loci for both species; 95°C for 15 min, followed by 35 cycles of 94°C for 30 s, 60°C for 90 s and 72°C for 60 s, followed by a final extension of 60°C for 30 min. Forward primers contained a fluorescent color of the G5 label set (FAM, VIC, NED or PET). One μl of diluted PCR product was added to 12 μl of GeneScan™ LIZ®500(‐250) size Standard (Applied Biosystems)/formamide, and PCR products were visualized on a Biosystems 3730 Sequencer (Applied Biosystems). Genotypes for 20 individuals from each population were scored using Genemapper™ v.3.7 (Applied Biosystems) software with amplification and fragment analysis repeated once for any individuals that failed to amplify or produce scorable bands in the first instance. Allele bins were manually assigned and automatically checked. Sequences containing the assayed microsatellites are available at the GenBank (www.ncbi.nlm.nih.gov/genbank) database under accession numbers MTO80657–MTO80688.

Utility of loci was assessed for each species. Departure from Hardy–Weinberg equilibrium (HWE) and Linkage Disequilibrium (LD) among locus pairs were tested using exact tests implemented in GENEPOP v4.2 (Raymond & Rousset, 1995). Frequency of null alleles and their effect on differentiation estimates was assessed with FREENA (Chapuis & Estoup, 2007). Significant (*p* < .0001 with Bonferroni correction applied) departure from HWE was found in 15 out of 352 locus/population combinations for *A*. *hilliana* and in nine out 240 locus/population combinations for *A*. *spondylophylla*, but never for more than four loci per population. There was potential evidence of null alleles at some locus/population combinations for both species. Null allele frequencies ranged from 0.00 to 0.376 for *A. hilliana* and from 0.00 to 0.365 for *A. spondylophylla* (Table S2). However, *F*_ST_ values were not significantly different in raw and corrected data sets (data not presented) so null alleles are likely to have little effect on related analyses (Chapuis & Estoup, 2007). Significant (*p* < .0001 with Bonferroni correction applied) LD was detected in 14 out of 120 locus combinations for *A*. *hilliana* and in two out of 120 locus combinations for *A*. *spondylophylla*.

### 2.6 Chloroplast and nuclear marker analysis

The number of haplotypes (*H*) and the number of private haplotypes (*H*p) detected in each population of each species were counted. Estimates of nucleotide and haplotype diversity including the total number of variable sites (*S*), total number of haplotypes (*h*), overall haplotype diversity (*H*d), nucleotide diversity (*π*), and parameters of neutrality tests that can changes in population size (Tajima’s *D*, Fu’s *F*s statistic and Ramos–Onsins and Rozas’ *R*_2_ test), were obtained for each species using D_NA_SP v6.12.03 (Librado & Rozas, 2009). Significance of values were assessed using a null distribution of 10,000 coalescent based simulations. Measures of phylogenetic diversity within populations (*h*_S_) and total diversity (*h*_T_), as well as coefficients of phylogenetic differentiation for unordered (*G*_ST_) and ordered alleles (*N*_ST_), were calculated with PERMUT v2.0 following Pons and Petit (1996). Phylogeographic structure was evaluated by assessing whether *N*_ST_ was significantly greater than *G*_ST_ using the U test for 1000 permutations in PERMUT.

Maximum parsimony phylogenetic networks (Polzin & Daneschmand, 2003) were constructed, and the shortest, least complex trees produced using the Median–Joining (MJ) network algorithm for multi‐state data (Bandelt et al., 1999), as implemented in NETWORK v5.0.1.1 (Fluxus Technology Ltd). Character states had equal weighting, *ε* was set to 0 and MJ networks were verified using the Reduced Median (RM) algorithm. No star contraction pre‐processing was applied. Haplotypes present in each population were mapped across species distributions.

For nuclear microsatellites, estimates of genetic diversity, allelic richness (*N*_R_), expected (*H*e) and observed (*H*o) heterozygosity, and proportion of polymorphic loci (%*P*), as well as the Fixation index (*F*_IS_), were calculated using MSA v4.05 (Dieringer & Schlötterer, 2003). The number of private alleles per population (*N*_AP_) was calculated using GenAlEx v6.5 (Peakall & Smouse, 2012). The Newman population of *A. spondylophylla* was highly differentiated from all other populations due to a large number of unique alleles in high frequency. It is suspected that this population represents the rare hybrid *Acacia adoxa* var. *adoxa* × *spondylophylla* that occurs at low frequencies in the Ophthalmia Range west of Newman and from near Nullagine where the two parent species are common (Maslin et al., 2010). As a result, the Newman population that was collected as *A. spondylophylla* was removed from further analyses of the nuclear and chloroplast genomes.

Genetic structure was investigated using the Bayesian assignment approach in structure v2.3.2.1 (Pritchard et al., 2000). Analysis used the admixture ancestry model with the assumption of correlated allele frequencies amongst samples given the likelihood of high population similarity due to gene flow (Falush et al., 2003). Known population locations were used as priors. A burn‐in period of 10,000 was applied with 100,000 MCMC replications to assess *K* values ranging from *K* = 1 to *K* = the number of populations plus one, with 10 iterations of each *K* value. The optimal *K* value was determined by identifying the maximum showing limited deviation for the heuristic model likelihood statistics L(*K*), and the maximum in Δ*K* (Evanno et al., 2005). Likelihood statistics were estimated with structure harvester v.0.6.93 (Earl & von Holt, 2012). Mean permuted proportion of membership (*Q*) values for all individuals were graphed and mapped for populations. Given the propensity of the *ad hoc* model likelihood statistic Δ*K* to identify *K* = 2 when further genetic structuring is present (Janes et al., 2017) we split the data set for *A. spondylophylla* placing each population into one of each of two initial clusters (assigned via population *Q* > 0.50 for either Cluster One or Cluster Two). We then conducted hierarchical structure analysis on each subcluster in the same manner as for the full data set.

Principal coordinates analysis (PCoA) was conducted based on pairwise individual genetic distances using a standardized data set for populations of each species in GenAlEx. Global and pairwise among population measures of genetic divergence including allelic fixation (*F*_ST_) and Jost’s estimate of allelic differentiation (*D*j, Jost, 2008) were calculated for each species with *p* values calculated on 999 permutations using GenAlEx. Hierarchical partitioning of population genetic structure was further characterized using the AMOVA method conducted on *F*_ST_ values at the population level using GenAlEx.

Mantel tests were used to test for the association of genetic differentiation (linearized *F*_ST_ (*F*_ST_/1−*F*_ST_)) with log geographic distance among populations. Significance tests were conducted using 999 permutations of sample site locations with all analyses conducted using GenAlEx.

Differences in chloroplast diversity (the number of haplotypes *H*) and nuclear diversity estimates (*N*_R_, *H*o, *He*) between species were assessed using single factor ANOVA without replication performed in R v3.6.2 (R Core Team, 2019). Analysis of differences in chloroplast (*H*) and nuclear (*N*_R_, *H*o, *H*e) diversity measures between the topographically complex Hamersley and Chichester Range populations and populations located in the surrounding lowlands were undertaken separately for each species using single factor ANOVA in R. Analysis of differences in chloroplast (*H*) and nuclear (*N*_R_, *Ho*, *He*) diversity measures with elevation (m) were also undertaken for each species using regression with bounded genetic diversity measures (*H*o and *H*e) arcsine transformed prior to analysis. The ratio of pollen to seed dispersal was calculated for each species following Ennos (1994) where Pollen: Seed = [*A*(1+*F*_IS_)−2*C*]/*C*, where *A* = (1/*F*_ST_)−1 and where *C* = (1/*G*_ST_)−1.

## 3 RESULTS

### 3.1 Chloroplast haplotype diversity

**FIGURE 2:**
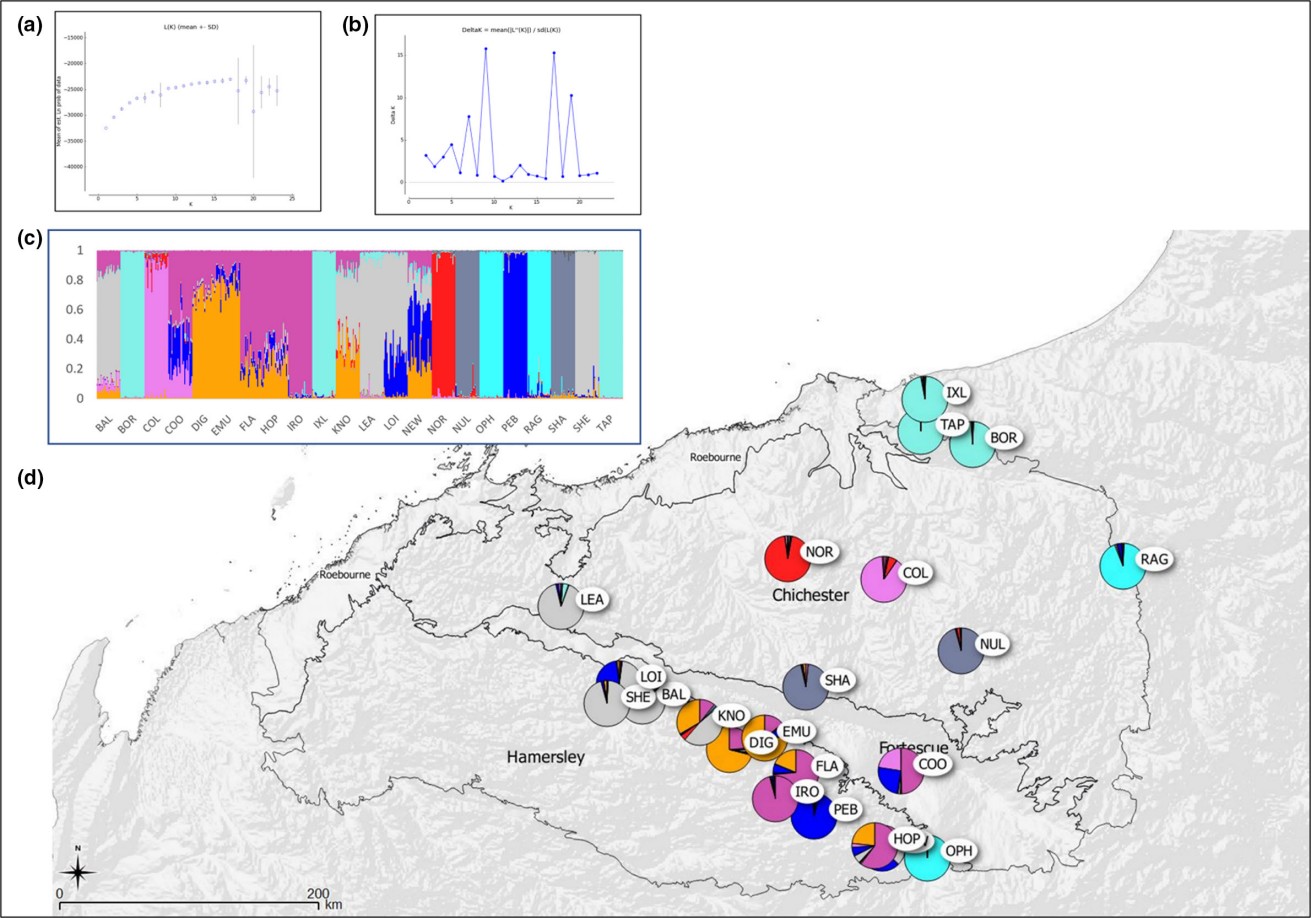
Assignment of individuals and populations to genetic clusters identified via Bayesian analysis of multilocus nuclear microsatellite genotype data for *Acacia hilliana* in the Pilbara bioregion. (a) Mean values of the *ad hoc* test statistic Ln probability of the data (L(*K*)). (b) Values of the *ad hoc* test statistic Delta *K* (Δ*K*). (c) Bar plot indicating the assignment of individuals and populations to one of nine genetic clusters. Each individual is represented as a vertical line partitioned into nine segments whose length is proportional to the individual coefficients of membership in each of nine clusters. Population codes on the *x*‐axis correspond to those in Table 1. (d) Geographic distribution of genetic clusters. Colors correspond to those in (c). Sections of pie charts show the proportion of assignment of the given population to each genetic subcluster. Labels correspond to population codes in Table 1

For *A. hilliana*, 2368 cpDNA sites were scored. There were 33 variable sites with 30 parsimony‐informative sites, leading to the identification of 25 haplotypes (Table 2). For *A*. *spondylophylla* 2108 sites were scored, with 15 variable sites and 15 parsimony‐informative sites leading to the identification of 12 haplotypes. Mean haplotype diversity and the mean number of private haplotypes per population were similar and low for each species but slightly greater for *A*. *hilliana* (Table 1); however, there was no statistically significant difference in the number of haplotypes (*H*) between species (Table 1, *F*_(1 (df between groups), 34 (df within groups))_ = 1.711, *p* < .199). *Acacia hilliana* had greater overall haplotype diversity (*H*d) and lower genetic differentiation among populations than *A*. *spondylophylla* (Table 2). There was little sharing of haplotypes among populations for either species (Figure 2). Haplotypes specific to populations were detected for 17 populations of *A. hilliana* and eight populations of *A*. *spondylophylla*. However, for both species, distinct haplotypes in the same population were on average more closely related than distinct haplotypes from different populations (*pN*_ST_ > *G*_ST_, Table 2). Neutrality tests were non‐significant, with the exception of the Fu’s *F*s test suggesting possible range expansion for *A*. *hilliana* (Table 2). The number of haplotypes (*H*) was not significantly greater in populations located on the ranges compared to populations located in the rest of the Pilbara for either species (*F*_(1, 20)_ = 1.03, *p* = .322 for *A. hilliana*; *F*_(1, 20)_ = 0.809, *p* = .386 for *A. spondylophylla*).

**TABLE 2:** Estimates of sequence diversity parameters, divergence parameters and neutrality tests for three and four chloroplast regions in *Acacia hilliana* and *Acacia spondylophylla* in populations from the Pilbara bioregion. Diversity and divergence parameters include total number of variable sites (*S*), the total number of haplotypes (*h*), overall haplotype diversity (*H*d), nucleotide diversity (*π*), genetic diversity within populations (*h*_S_) total genetic diversity (*h*_T_), coefficients of genetic differentiation for unordered (*G*_ST_) and ordered alleles (*N*_ST_) and mean permuted value of *N*_ST_ (*pN*_ST_). Parameters of neutrality tests include Tajima's *D* (*D*), Fu’s *F*s statistic (*F*s) and Ramos‐Onsins and Rozas’ *R*_2_ statistic (*R*_2_). Standard deviation (*H*d, *π*), standard error (*H*_s_, *H*_T_, *G*_ST_, *N*_ST_), or *p*‐value (*pN*_ST_, *D*, *F*s, *R*_2_) in parentheses

Species	*S*	*h*	*H*d	*π*	*h*_S_	*h*_T_	*G*_ST_	*N*_ST_	*pN*_ST_>G_ST_	*D*	*F*s	*R*_2_
*Acacia hilliana*	33	25	0.935 (0.008)	0.0016 (0.000)	0.164 (0.049)	0.897 (0.020)	0.831 (0.051)	0.897 (0.044)	<.010	−1.187 (>0.100)	−6.772 (0.001)	0.0510 (0.151)
*Acacia spondylophylla*	15	12	0.873 (0.016)	0.0013 (0.000)	0.094 (0.053)	0.926 (0.041)	0.898 (0.057)	0.953 (0.029)	<.010	−0.161 (>0.100)	−0.612 (0.120)	0.0889 (0.517)

Visual investigation of haplotype networks indicated simple star‐like patterns with the presence of two common haplotypes central to the network in each species. Haplotypes central to the network were not always located at the geographic center of the sampling area. For *A. hilliana*, two dominant haplotypes with central positions within the network (H10 and H11) were present in a number of central (KNO, EMU, FLA), southern (PEB, HOP) and far north‐eastern populations (IXL, RAG). However, the most divergent haplotypes (H1, H4 and H5) were also located in central (BAL) and north‐eastern populations (BOR, Figure 1a). For *A*. *spondylophylla*, the most common central haplotype (H6) was present in central populations (LOI, SOL, MUL and WHI) and another common haplotype with a central position within the network (H1) was present at both the most north‐eastern (BAT) and the most western populations (PAN, Figure 1b).

### 3.2 Nuclear diversity and differentiation

Nuclear microsatellite diversity was moderate for both species (Table 1). Diversity estimates (*N*_R_, *H*o, *H*e) were all significantly greater for *A. hilliana* (Table 1, *F*_(1, 34)_ = 38.869, *p* < .000; *F*_(1, 34)_ = 17.634, *p* < .000; *F*_(1, 34)_ = 26.856, *p* < .000). Observed heterozygosity was lower than expected heterozygosity at most populations for each species. Although fixation indices varied among populations, mean values were low and generally positive for both species (Table 1).

For *A. hilliana*, initial structure analysis revealed leveling of L(*K*) at *K* = 6 and at *K* = 8 although with some standard deviation, and further leveling off at *K* = 9 with no deviation (Figure 2a). Although a number of other peaks were observed, Δ*K* showed the greatest peak at *K* = 9 (Δ*K* = 16, Figure 2b). Mean permuted *Q* values obtained via structure analysis for all individuals at *K* = 9 are graphed (Figure 2c) and populations mapped (Figure 2d). In this analysis, populations along the Hamersley Range, except for OPH, and including LEA on the Chichester Range and COO in the Fortescue Valley showed a degree of admixture. These populations can be differentiated into western (LEA, SHE, LOI, and BAL) and eastern (COO, DIG, EMU, FLA, HOP, IRO, NEW, and PEB) groups, with significant admixture in the central KNO population (Figure 2d). OPH and remaining populations extending northeast from the Hamersley Range and Fortescue Valley were more distinct. The northernmost populations BOR, IXL, and TAP grouped together, as did the central NUL and SHA populations, while central COL and NOR populations were largely distinct from all others. Despite their separation latitudinally, the two most easterly populations OPH and RAG grouped together.

**FIGURE 3:**
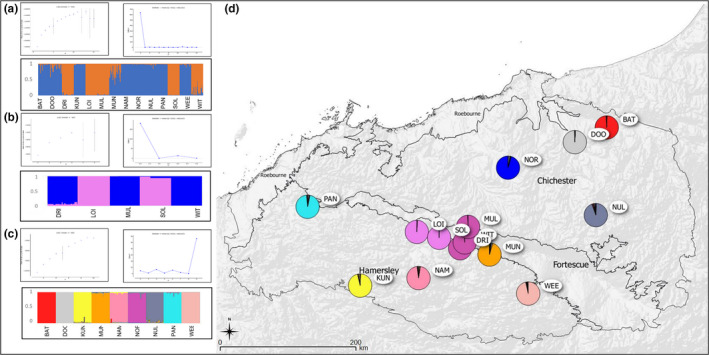
Assignment of individuals and populations to genetic clusters identified via Bayesian analysis of multilocus nuclear microsatellite genotype data for *Acacia spondylophylla* in the Pilbara bioregion. (a) Mean values of the *ad hoc* test statistic ln probability of the data (L(*K*)), values of the *ad hoc* test statistic Delta*K* (Δ*K*), and bar plot indicating the assignment of individuals and populations to one of two optimally determined genetic clusters. Each individual is represented as a vertical line partitioned into two segments whose length is proportional to the individual coefficients of membership in each of two clusters. (b) Hierarchical clustering analysis of populations from Cluster One (Orange) in (a). Mean values of the *ad hoc* test statistic ln probability of the data (L(*K*)), values of the *ad hoc* test statistic Delta*K* (Δ*K*), and bar plot indicating the assignment of individuals and populations to one of two optimally determined genetic clusters. Each individual is represented as a vertical line partitioned into two segments whose length is proportional to the individual coefficients of membership in each of two clusters. (c) Hierarchical clustering analysis of populations from Cluster Two (Blue) in (a). Mean values of the *ad hoc* test statistic ln probability of the data (*L*(*K*)), values of the *ad hoc* test statistic Delta*K* (Δ*K*), and bar plot indicating the assignment of individuals and populations to one of nine optimally determined genetic clusters. Each individual is represented as a vertical line partitioned into nine segments whose length is proportional to the individual coefficients of membership in each of nine clusters. (d) Geographic distribution of eleven genetic clusters from the hierarchical analysis. Colors correspond to those in (b) and (c). Sections of pie charts show the proportion of assignment of the given population to each genetic subcluster. All population codes correspond to those in Table 1

In structure analysis of *A. spondylophylla*, L(*K*) increased steadily leveling off at *K* = 5 but with a large standard deviation, then continued to increase leveling off with no significant deviation at *K* = 11 (Figure 3a). Δ*K* showed the most significant peak at *K* = 2 (Δ*K* = 638, Figure 3a). Individual *Q* values for the two subclusters (*K* = 2) are indicated (Figure 3a). Cluster One consisted of five centrally located populations (DRI, LOI, MUL, SOL, and WIT), while Cluster Two encompassed all more peripherally located populations (BAT, PAN, DOO, KUN, MUN, NAM, NOR, NUL, and WEE, Figure 3a). There was limited admixture between the two clusters, except at MUN. Results of hierarchical analysis of each of these primary clusters is as follows. For Cluster One, L(*K*) increased steadily leveling off at *K* = 4 with little significant deviation (Figure 3b) and Δ*K* showed the most significant peak at *K* = 2 (Δ*K* = 181, Figure 3b). Individual *Q* values for Cluster One when resolved into two subclusters (i.e., *K* = 2), are indicated (Figure 3b). For Cluster Two, L(*K*) increased steadily leveling off at *K* = 4 with no significant deviation (Figure 3c) and Δ*K* showed the most significant peak at *K* = 9 (Δ*K* = 56, Figure 3c). Individual *Q* values for Cluster Two when resolved into nine subclusters (*K* = 9) are indicated (Figure 3c). Population membership values from the hierarchical analysis, that is, final number of genetic clusters *K* = 11, are mapped (Figure 3j). In a geographic context, hierarchical analysis of Cluster One separated populations DRI, MUL, and WIT from populations LOI and SOL, with very little admixture (Figure 3d) while all peripheral populations from Cluster Two were identified as separate populations or subclusters (Figure 3d).

**FIGURE 4:**
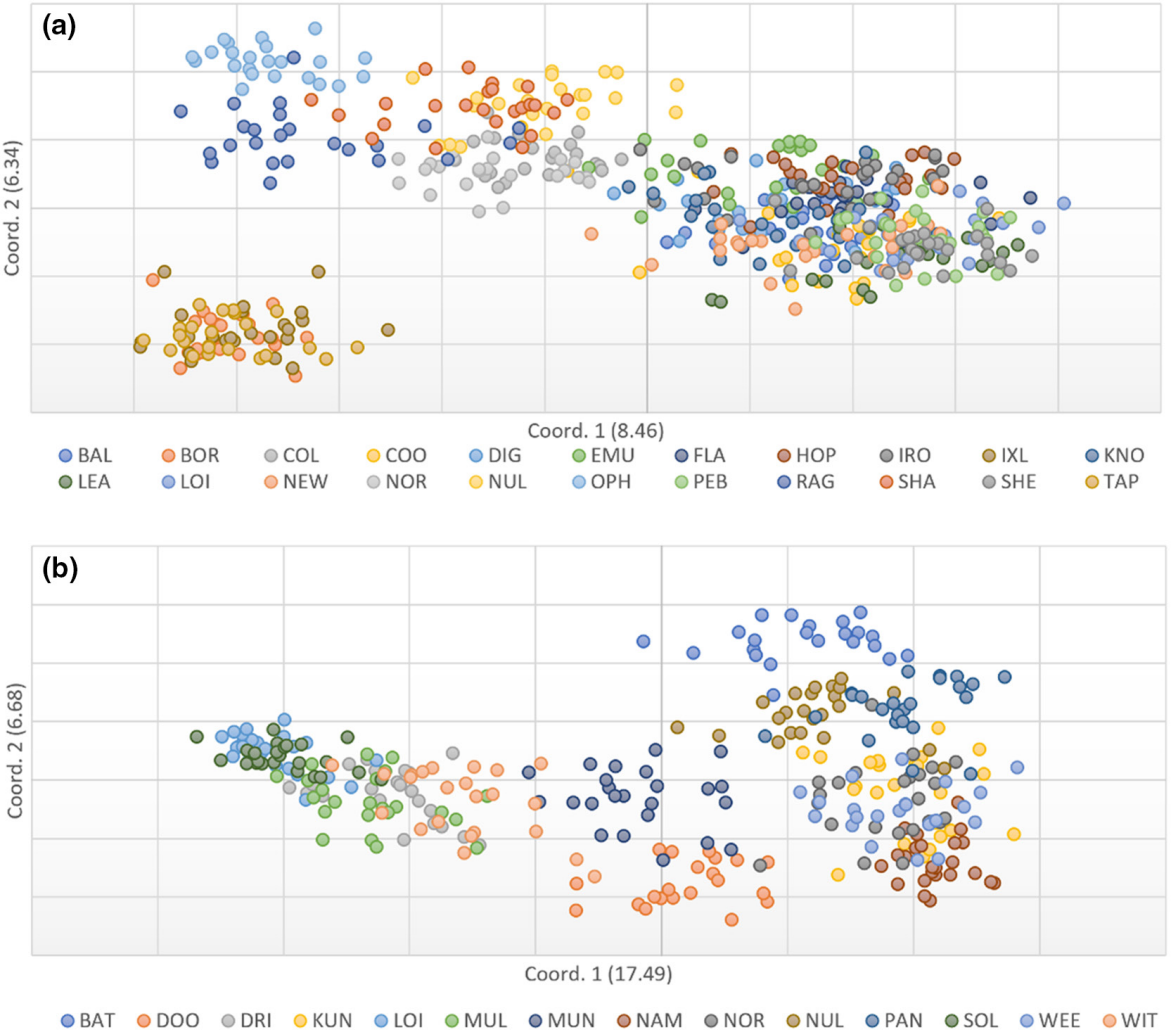
Principal co‐ordinates analysis of the genetic distance between sampled individuals of (a) *Acacia hilliana* and (b) *Acacia spondylophylla* in the Pilbara bioregion. Population codes correspond to those in Table 1

The principal co‐ordinates analyses indicate patterns of genetic structure for the first two principal co‐ordinates of each species (Figure 4). The first two axes in *A. hilliana* separated the northernmost populations BOR, IXL and TAP from all remaining populations, and there was some separation of the easterly OPH and RAG populations, the central NUL and SHA populations, and to a lesser extent the central COL and NOR populations (Figure 4a). The first two axes explained 14.80% of the total variation in *A. hilliana*. The first two axes in *A. spondylophylla* illustrate some separation among more central populations LOI, MUL, SOL, and DRI, and to a lesser extent the central MUN and WIT populations, from other more peripheral populations (Figure 4b). The first two axes explained 24.17% of the total genetic variation in *A. spondylophylla*.

**FIGURE 5:**
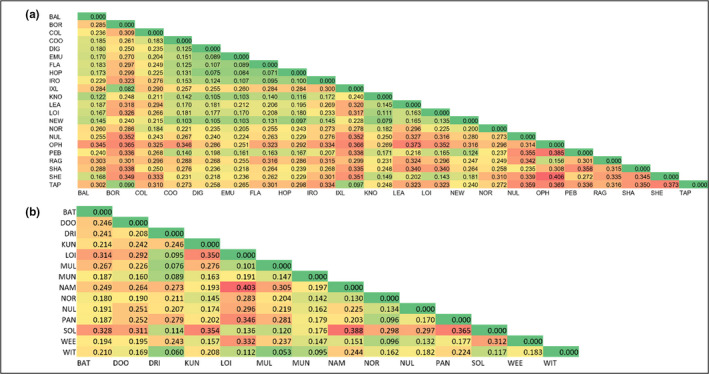
Heatmaps of pairwise population genetic divergence (*F*_ST_) for nuclear microsatellite data for (a) *Acacia hilliana* and (b) *Acacia spondylophylla* in the Pilbara bioregion. Population codes correspond to those in Table 1

Measures of genetic divergence among populations were high for each species. Genetic fixation among populations was lower for *A. hilliana* (global *F*_ST_ = 0.260, *p* < .001) than for *A. spondylophylla* (global *F*_ST_ = 0.349, *p* < .001), although allelic differentiation among populations was greater for *A. hilliana* (*D*_ST_ = 0.514, *p* < .001) than for *A. spondylophylla* (*D*_ST_ 0.448 *p* < .001). Pairwise values of fixation (*F*_ST_) among populations were variable, ranging from 0.071 to 0.406 for *A. hilliana* (Figure 5a) and from 0.053 to 0.403 for *A. spondylophylla* (Figure 5b).

AMOVA revealed that each species had the greatest degree of genetic diversity partitioned within populations. The degree of genetic diversity partitioned among populations was lower for *A*. *hilliana* (36%) compared to *A*. *spondylophylla* (49%).

There was a significant positive correlation between geographic distance and genetic differentiation across the sampled region for *A. hilliana* (*R* ^2^ = .322, *p* = .001). There was a weak but significant positive correlation across the sampled region for *A. spondylophylla* (*R* ^2^ = .126, *p* = .008).

Significantly greater contemporary diversity (*He*) was observed in populations of *A. hilliana* located on the ranges compared to populations located in the rest of the Pilbara (*F*_(1, 20)_ = 4.53, *p* = .046), and values of *Ho* and *He* were significantly greater for *A. hilliana* populations with higher elevation (*R* ^2^ = .346, *p* = .004 and *R* ^2^ = .295, *p* = .009). Pollen to seed dispersal ratios were moderate; 12.1:1 for *A. hilliana* and 15.6:1 for *A. spondylophylla*.

## 4 DISCUSSION

Comparative analysis of congeneric species provides a phylogenetically controlled evaluation of how taxon‐specific life history and ecological traits shape genetic diversity and connectivity. Our analysis of two *Acacia* species showed levels of genetic diversity comparable to those of other co‐distributed, widespread *Acacia* species in the arid Pilbara landscape, confirming expectations of the effect of species range on levels of genetic diversity. Evaluation showing greater phylogeographic structure for the two low‐statured study species, compared to other Pilbara *Acacia* species that are either large shrubs or trees, was consistent with the predicted association of stature with limited seed dispersal, as well as limited pollen dispersal. Also, as predicted, while patterns of genetic structure were largely concordant at the landscape scale, more subtle differences in diversity, differentiation and demography were evident and consistent with differences in the life history traits of these species.

### 4.1 Patterns of diversity and phylogeographic structure

Both haplotype and nuclear diversity in *A*. *hilliana* and *A*. *spondylophylla* were comparable to those found for two other widespread *Acacia* species with populations in the Pilbara bioregion, *A. ancistrocarpa* (Levy et al., 2016) and *A. pruinocarpa* (Nistelberger et al., 2020). This result supports our first prediction regarding genetic diversity and is consistent with population genetic theory that plant species with widespread ranges maintain greater diversity than species with restricted range sizes, as they are expected to be less impacted overall by stochastic environmental and genetic effects that may negatively affect population sizes (Allendorf et al., 2013; Ellstrand & Ellam, 1993; Godt & Hamrick, 1993). The levels of haplotype and nuclear diversity in these widespread *Acacia* contrasts with lower haplotype and nuclear diversity detected for the range limited and near‐endemic *A*. *atkinsiana* (Levy et al., 2016). Range size or distribution is known to have a strong effect on genetic diversity of the Australian flora (Broadhurst et al., 2017). These results confirm a significant influence of species geographic range on the maintenance of genetic diversity over both macro‐ and micro‐evolutionary temporal scales in this arid landscape.

Detection of significant patterns of phylogeographic structure also matched our predictions. Seed dispersal is a key life history trait affecting gene flow among populations, with significant phylogeographic structure in chloroplast haplotypes indicating historical limitations to gene flow via seed dispersal. This would be expected for *Acacia* in the arid Pilbara, as primary dispersal of seeds is by gravity and secondary dispersal by myrmecochory. Gravity dispersal is likely to result in short distance primary seed dispersal of a few meters, while longer distance dispersal events are possible via ant dispersal (a few hundred meters, Pascov et al., 2015), although ant dispersed species have also shown some of the shortest dispersal distances documented, of just a few meters (Gómez & Espadaler, 1998; Hughes et al., 1994; Hughes & Westoby, 1992). For species that lack longer distance seed dispersal mechanisms, significant limitations to seed dispersal are most strongly associated with stature, with species of low stature having more restricted seed dispersal distances (Duminil et al., 2009; Nybom, 2004; Thomson et al., 2011). Our result of more pronounced phylogeographic structure in *A. hilliana* and *A. spondylophylla* than that found for populations of both the widespread large (to 4 m) shrub *A. ancistrocarpa* (*G*_ST_ = 0.654, Levy et al., 2016) and the tall (to 12 m) tree species *A. pruinocarpa* (*G*_ST_ = 0.230, Nistelberger et al., 2020), is consistent with this prediction and suggests that growth form or stature is a key life history trait driving seed dispersal distance in these *Acacia*. The lack of detectable phylogeographic structure found in *A*. *atkinsiana* (Levy et al., 2016), which is also a medium sized (to 3.5 m) shrub but with more restricted distribution, is more likely to be associated with the maintenance of a very limited number of extant haplotypes (four) over a shorter geographic range.

While we observed broad concordance in genetic patterns with our study species following predictions based on distribution and stature, we also observed differences between them in terms of contemporary genetic diversity and structure, indicating effects of other variable life history and ecological traits. For example, within population diversity was significantly lower and global and pairwise population measures of genetic differentiation were greater for *A. spondylophylla* than for *A. hilliana*. These differences are consistent with population genetic theory (Allendorf et al., 2013; Ellstrand & Ellam, 1993; Ghazoul, 2005; Leimu et al., 2010; Petit & Hampe, 2006) and our prediction of reduced gene flow among populations of *A. spondylophylla*, which has a greater degree of geographic population disjunction, lower localized population density, smaller population size, shorter individual longevity, and lower fecundity, than *A. hilliana*.

Genetic differentiation among populations located on the Hamersley and Chichester Ranges was further pronounced for *A. spondylophylla*, where range populations are less continuous, less dense, and smaller than those of *A. hilliana* (Maslin et al., 2010). A role of geographic population connectivity, local population density and population size in maintaining contemporary population diversity and genetic connectivity is also suggested by significantly greater genetic diversity (*H*e) in populations of *A. hilliana* located on the Hamersley and Chichester Ranges compared to populations located in the rest of the Pilbara, and significantly greater diversity (*H*e and *H*o) for *A. hilliana* populations with elevation.

### 4.2 Limited contemporary genetic connectivity

Interestingly, our results of genetic differentiation in *A*. *hilliana* and *A*. *spondylophylla* were not consistent with our prediction of genetic connectivity via contemporary gene flow being comparable to that of other widespread *Acacia* in the Pilbara. Values of genetic differentiation for *A*. *hilliana* and *A*. *spondylophylla* (*F*_ST_ = 0.260 and 0.349, respectively) were much greater than those reported for Pilbara populations of the widespread *A. ancistrocarpa* (*F*_ST_ = 0.047, Levy et al., 2016) and *A. pruinocarpa* (*F*_ST_ = 0.062, Nistelberger et al., 2020), and even for the range restricted *A. atkinsiana* (*F*_ST_ = 0.190, Levy et al., 2016). Unexpectedly high measures of nuclear genetic differentiation indicate strong barriers to pollen as well as seed dispersal on more microevolutionary spatial temporal scales. Genetic structure of the nuclear genome is known to be most strongly influenced by the mating system (Duminil et al., 2007) and mechanisms of pollen and seed dispersal. Whilst *Acacia* typically have predominantly outcrossed mating systems (Broadhurst et al., 2008; George et al., 2008; Millar et al., 2008, 2019; Ng et al., 2009), substantial levels of selfing have also been recorded (Coates et al., 2006; Mandal et al., 1994). Our results may be explained by increased levels of selfing or bi‐parental inbreeding (mating among related individuals) although reported *F*_IS_ values do not differ greatly between the study species and other Pilbara *Acacia*. It is possible that *A. hilliana* and *A. spondylophylla* could have more specialized insect pollinators with more limited dispersal abilities, or there could be unrecognized barriers between populations that limit the movement of important pollinators. Alternatively, it may be that stature of individual plants plays a more important role than currently recognized in long distance or localized pollen dispersal, in addition to seed dispersal, for generalist insect pollinated species in arid landscapes.

Pollen: seed dispersal ratios for *A. hilliana* and *A. spondylophylla* are most similar to those observed for the taller shrub species *A. atkinsiana* (12.5:1, unpublished data but see Levy et al., 2016). However, the relative contributions of pollen and seed dispersal to overall genetic connectivity in *Acacia* in the Pilbara vary considerably even for tall shrub and tree species. Much higher values (43.5:1 for the tall shrub *A. ancistrocarpa*, unpublished data but see Levy et al., 2016), as well as much lower values (3:1 for the tall tree *A. pruinocarpa*, Nistelberger et al., 2020) have also been observed. Variability in pollen: seed dispersal ratios suggest that taxon‐specific life history and ecological traits other than mating system, pollen and seed dispersal syndromes, and stature also play key roles in driving diversity, differentiation, and demography. *Acacia ancistrocarpa* occurs in rather continuous populations at moderate density, which would theoretically facilitate greater levels of pollen dispersal (Aguilar et al., 2006; Ghazoul, 2005; Leimu et al., 2006), while *A. pruinocarpa* populations are more disjunct and individual trees within populations tend to be more widely spaced with possibly more limited opportunity for connectivity via pollen dispersal. More direct studies are required to confirm mating system and methods of pollen and seed dispersal in Pilbara *Acacia* to be able to explain this apparent variation in the relative contributions of pollen and seed dispersal between species.

### 4.3 Landscape features and genetic structure

Ranges have been hypothesized to have acted as historical refugia in arid landscapes (Byrne et al., 2008) and there was evidence of the elevated topographically complex Hamersley and Chichester Ranges acting as refugia in *Eucalyptus leucophloia* subsp. *leucophloia* (Byrne et al., 2017). However, there was little support for this hypothesis in *A. hilliana* and *A. spondylophylla*, with no evidence for increased haplotype diversity on the Hamersley and Chichester Ranges, nor reduced diversity or signal of expansion off the ranges. Instead, low to moderate diversity and shallow divergence among extant haplotypes, along with simple star‐like networks and phylogeographic structure, support a hypothesis of relatively stable demographic history and localized population persistence both on and off the ranges. This result is concordant with scenarios of historical isolation and persistence for extant populations of other *Acacia* species within the Pilbara (Levy et al., 2016; Nistelberger et al., 2020). Simple star‐like networks have been associated with historical population persistence and a lack of demographic signal for *A. atkinsiana* (Levy et al., 2016), and, while haplotype diversity and network complexity are greater, patterns also indicate historical population isolation and persistence for *A*. *ancistrocarpa* (Levy et al., 2016) and *A*. *pruinocarpa* (Nistelberger et al., 2020). A notable exception is a shared haplotype (H1) of *A. spondylophylla* that occurs in single populations at the north‐eastern (BAT) and north‐western (PAN) geographical extremes. This may be a result of homoplasy rather than long distance seed dispersal, and a similar incidence of a geographically disjunct haplotype has been noted in *A. ancistrocarpa*, which has a similar population range within the Pilbara (Levy et al., 2016).

Although there is general support for localized population persistence of Pilbara *Acacia*, greater haplotype diversity and lower phylogenetic divergence, along with a significant test of non‐neutrality in extant populations, provides some evidence for historical range expansion for *A. hilliana*. *Acacia hilliana* does have greater individual longevity (50+ years vs 5–15 years) and fecundity than *A. spondylophylla* (Maslin et al., 2010). Greater longevity may provide greater potential for expansion by providing greater opportunity for spatial and temporal seed dispersal. A more complex network of dispersal and significant test of non‐neutrality, also suggesting a scenario of population expansion have been observed for the tall tree species, *A. pruinocarpa*, in the same landscape (Nistelberger et al., 2020). *Acacia pruinocarpa* is also long lived (50+ years) and sparsely scattered (Maslin et al., 2010), and these traits would also provide increased opportunity for spatial and temporal dispersal of pollen and seed.

Although the ranges do not appear to have had a role as historical refugia there is evidence for different patterns of contemporary genetic structure associated with these features in *A. hilliana* and *A. spondylophylla*. Primary genetic clusters located on the Hamersley and Chichester Ranges (*A. hilliana*) or centrally located populations also largely on these ranges (*A. spondylophylla*), were differentiated from more peripheral populations at lower elevation. Limited admixture and high measures of differentiation indicate limited contemporary gene flow between these differing landscapes. Significantly greater contemporary diversity was also observed in populations located on the ranges compared to populations located in the rest of the Pilbara. A similar pattern was found for the near Pilbara endemic *A. atkinsiana* (Levy et al., 2016), with populations on the Hamersley and Chichester Ranges showing a greater degree of genetic connectivity and being differentiated from near coastal and other peripheral populations that occur at lower elevation. However, these patterns may be more related to taxon‐specific ecological traits regarding geography and demography rather than elevation or the topographical complexity of the ranges *per se*. Extant populations located on the Hamersley and Chichester Ranges are less geographically disjunct and of greater local density than populations located off the ranges for all three species. Thus, the landscape scale patterns of genetic structure and diversity seen in both study species are consistent with predictions of population genetic theory that associate restricted gene flow, increased genetic differentiation and reduced diversity with geographical disjunction and low population density (Allendorf et al., 2013; Ellstrand & Ellam, 1993; Godt & Hamrick, 1993). The results strongly support a recent meta‐analysis that has shown degree of population disjunction to be the greatest driver of microevolutionary population genetic differentiation in the Australian flora (Broadhurst et al., 2017).

## 5 CONCLUSION

Our comparative, trait‐based approach suggests complex contributions of taxon‐specific life history and ecological traits including species range, individual stature, and the degree of geographic disjunction in shaping genetic diversity and structure at both macroevolutionary and microevolutionary spatial and temporal scales in a common landscape. Analysis in co‐distributed congeneric species provides a phylogenetically controlled evaluation of the effects of life history and ecological traits that highlights the influence of stature on genetic connectivity via both pollen and seed dispersal, a factor that has not often been recognized, as well as the effect of geographic disjunction on population genetic structure. There was little evidence for topographically complex elevated ranges acting as major historical refugia for *Acacia* in this landscape and the influences of life history traits are evident against a background of historical isolation and persistence of extant populations of *Acacia* of the arid Pilbara. Integrative studies that investigate the ecology of pollen and seed dispersal could further contribute to refining hypotheses regarding the roles of stature and localized population density in maintaining demographic and genetic connectivity in key species in arid landscapes, and other highly structured landscapes globally.

## 6 AUTHOR CONTRIBUTIONS

**Melissa Ann Millar:** Data curation (supporting); formal analysis (equal); writing – original draft (lead); writing – review and editing (lead). **Rachel Maria Binks:** Data curation (lead); formal analysis (equal); methodology (equal); writing – review and editing (supporting). **Sarah‐Louise Tapper:** Data curation (supporting); formal analysis (equal). **Bronwyn Macdonald:** Data curation (supporting); formal analysis (supporting); resources (supporting). **Shelley McArthur:** Resources (supporting). **Margaret Hankinson:** Data curation (supporting); formal analysis (supporting). **David J. Coates:** Resources (equal); writing – review and editing (supporting). **Stephen vanLeeuwen:** Conceptualization (equal); funding acquisition (lead); project administration (supporting); resources (equal); writing – review and editing (supporting). **Margaret Byrne:** Conceptualization (equal); methodology (lead); project administration (lead); resources (equal); writing – review and editing (supporting).

## 7 CONFLICT OF INTEREST

The authors declare no conflict of interest.

8

## Supporting information


Table S1.
Table S2.

## Data Availability

Chloroplast sequences: Genbank accessions MW138725‐MW138756 and MW696776‐MW696791. Microsatellite containing nuclear sequences: Genbank accessions MTO80657‐MTO80688. Haplotype and genotype data: Mendeley Data, https://doi.org/10.17632/67r5zxhd59.1.
