# Supplementary material for: Limited phylogeographic and genetic connectivity in Acacia species of low stature in an arid landscape
Source: Ecol Evol. 2022 Jul 6;12(7):e9052. doi: 10.1002/ece3.9052 (PMC9257521; doi:10.1002/ece3.9052)
Supplement: Supplementary file 1 — Table S1. Table S2. [file ECE3-12-e9052-s001.docx]

**Supporting Information**

**Table S1.** Characterisation of 16 microsatellite loci developed for *Acacia hilliana* and 16 microsatellite loci developed for *Acacia spondylophylla*. Details are provided for locus name, primer sequence, GenBank accession number, repeat motif and the size range of observed alleles in base pairs for adult individuals across sampled populations in this study.

| ***Acacia hilliana*** | |  |  |  |
| --- | --- | --- | --- | --- |
| **Locus** | **Primer sequence (5′-3′)** | **GenBank accession no.** | **Repeat motif** | **Allele size range (bp)** |
| Ah04 | F:GGCGTGAAAGTCTTGATGGT R:CACCATCTTCATCATCGCAG | MT080657 | (AG)_11_ | 84-109 |
| Ah06 | F:ACAGACACCAAATCACGCAG R:TGTTGAAGGATTAGGCCCAG | MT080658 | (AAT)_15_ | 72-135 |
| Ah10 | F:CACCATGCAATCAATGGTTC R:GAGTAATGTCGAAGTCGAGCG | MT080659 | (AG)_11_ | 135-187 |
| Ah15 | F:ACAATAGTGGGACGTGGACC R:TGACCCTGAAACGTCAAGTG | MT080660 | (AG)_18_ | 154-190 |
| Ah16 | F:CCATCCAAGTCACAGTTCCA R:AGGCACTTGGCAAATTAAGC | MT080661 | (AG)_8_ | 175-199 |
| Ah21 | F:GACTGCCGATGGAGGTGTAT R:TTTGCTCTTGCAGCTTCAGT | MT080662 | (AC)_10_ | 228-252 |
| Ah23 | F:CCACCATTTGCATCATGTTC R:TTGCTGCAGGTAGAGAAGCA | MT080663 | (AG)_19_ | 249-289 |
| Ah30 | F:GGATCTAGGTTTAGAAGCAAGCA R:TCTCTAACCTGCCCAACACA | MT080664 | (AG)_8_ | 110-122 |
| Ah38 | F:TCTTATTGTTGCCGGTTCAA R:CCCATGTAGGATGGAGTGTTC | MT080665 | (AG)_8_ | 147-192 |
| Ah49 | F:TCAGTGTTTGGACTCCACTG R:TGTTGGAAGAGATAGCCACAAA | MT080666 | (AG)_9_ | 88-112 |
| Ah50 | F:AAATCTGACCCAACAAGTCTTTA R:TCATTCAAGTCAGCCTTTAACCT | MT080667 | (AG)_9_ | 99-117 |
| Ah52 | F:AGGGTGAGTTCAAGAGGGAA  R:TGCCTTGGTTCTTTGTAGGA | MT080668 | (AG)_9_ | 102-130 |
| Ah57 | F:TCATCAAATCCTGACCGTTG R:TCTCGATTAAGAGGAAATCGG | MT080669 | (AG)_10_ | 119-139 |
| Ah58 | F:AAGGACGGTGAAAGAATTGG R:GGCCATCCCAATTAGAACATT | MT080670 | (AG)_10_ | 127-150 |
| Ah63 | F:GGTTCAAACCTGAGCCTGTT R:CAACCATTTGCAATGCACTAA | MT080671 | (ATC)_9_ | 134-159 |
| Ah66 | F:CAAGAAATGGACTGAAGTCAAGC R:CCTCAGGTCATCTTCAACTATACG | MT080672 | (AC)_10_ | 154-178 |
| ***Acacia spondylophylla*** | |  |  |  |
| **Locus** | **Primer sequence (5′-3′)** | **GenBank accession no.** | **Repeat motif** | **Allele size range (bp)** |
| As02 | F:GCCGTGAGAAGGTAACCAAA  R:TATCACTTGGGTGGCAGTTG | MT080673 | (AC)10 | 86-107 |
| As05 | F:AAGTGAGATTCGCCATCGAG  R:AGACACTCTTGCCTTCACCG | MT080674 | (AG)8 | 96-110 |
| As09 | F:TCGTGTTGTTCTTGGTGGTT  R:TTCGCAGGTTCACATCATTC | MT080675 | (AG)11 | 126-148 |
| As14 | F:AAGACGTGCAAGTCGTTGAA  R:CACCTGTCGGAGCAAGAAAT | MT080676 | (AC)10 | 164-178 |
| As15 | F:CCACCATCCACATCAATCAA  R:GCAACCTCTGGCTTATCCTG | MT080677 | (AG)17 | 153-187 |
| As20 | F:CGAACCGAGTCTGAACCATT  R:CTGCTTTGTCAAGAATGGCA | MT080678 | (AC)10 | 195-205 |
| As21 | F:TAGAGGAGAATCCCGCCAC  R:CCACTCCCATTCTTTACGGA | MT080679 | (AG)15 | 180-212 |
| As23 | F:AGCCGACCAATTTCTTTCAG  R:CATGCATGAATCACATTCCA | MT080680 | (AAT)16 | 194-224 |
| As24 | F:CTAGAACGGAGGCGTCAACT  R:TGCTCTTGCTTCCAAAGGTT | MT080681 | (AG)9 | 294-312 |
| As26 | F:TGGAATCTGAGAAGGCACAA  R:AGCGGTGTTGATCTGTTTCC | MT080682 | (AC)10 | 82-102 |
| As28 | F:TCCCTTTCTTTAAGCCGTGA  R:GTCACATTCGACTCCCACG | MT080683 | (AC)12 | 89-99 |
| As34 | F:GGGCCACAAGAGCTTATGAT  R:CCTACTGGCTCGACCTTCAA | MT080684 | (AAT)8 | 114-127 |
| As35 | F:TTCAATGGCAAGTTGCTCTG  R:TTAATGGTACCTTGGGCTCC | MT080685 | (AG)9 | 121-141 |
| As39 | F:GTCAAATGCAAATCAAAGGC  R:TGGACCAAGTTCTCATTCCC | MT080686 | (AC)8 | 138-167 |
| As42 | F:TTTGTCCCTCCCAATAAAGG  R:AAAGGCATCTTCATTGCCTG | MT080687 | (AG)12 | 135-158 |
| As51 | F:CGGATCTACCGATTCAGACAA  R:TACCGAGTCGATCATCCCAT | MT080688 | (AC)10 | 187-189 |

**Table S2.** Null allele frequencies of 16 microsatellite loci developed for *Acacia hilliana* and 16 microsatellite loci developed for *Acacia spondylophylla*. SE; standard error.

| Population | Locus |  |  |  |  |  |  |  |  |  |  |  |  |  |  |  |  |  |
| --- | --- | --- | --- | --- | --- | --- | --- | --- | --- | --- | --- | --- | --- | --- | --- | --- | --- | --- |
| *Acacia hilliana* | AH04 | AH10 | AH16 | AH30 | AH06 | AH15 | AH23 | AH57 | AH21 | AH38 | AH49 | AH50 | AH58 | AH52 | AH63 | AH66 | Mean | SE |
| BAL | 0.328 | 0.000 | 0.000 | 0.000 | 0.155 | 0.076 | 0.073 | 0.001 | 0.138 | 0.000 | 0.117 | 0.000 | 0.045 | 0.025 | 0.157 | 0.018 | 0.071 | 0.023 |
| BOR | 0.099 | 0.000 | 0.000 | 0.001 | 0.000 | 0.019 | 0.000 | 0.001 | 0.000 | 0.060 | 0.271 | 0.000 | 0.101 | 0.007 | 0.136 | 0.022 | 0.045 | 0.019 |
| COL | 0.376 | 0.000 | 0.048 | 0.000 | 0.000 | 0.000 | 0.026 | 0.001 | 0.000 | 0.000 | 0.123 | 0.016 | 0.128 | 0.000 | 0.000 | 0.071 | 0.049 | 0.024 |
| COO | 0.003 | 0.017 | 0.165 | 0.000 | 0.050 | 0.022 | 0.000 | 0.149 | 0.000 | 0.043 | 0.009 | 0.046 | 0.106 | 0.000 | 0.004 | 0.069 | 0.043 | 0.013 |
| DIG | 0.000 | 0.139 | 0.000 | 0.000 | 0.000 | 0.000 | 0.000 | 0.000 | 0.032 | 0.039 | 0.000 | 0.000 | 0.055 | 0.074 | 0.011 | 0.000 | 0.022 | 0.010 |
| EMU | 0.052 | 0.099 | 0.000 | 0.000 | 0.009 | 0.000 | 0.007 | 0.015 | 0.069 | 0.089 | 0.001 | 0.000 | 0.063 | 0.062 | 0.115 | 0.077 | 0.041 | 0.010 |
| FLA | 0.000 | 0.008 | 0.000 | 0.024 | 0.000 | 0.088 | 0.000 | 0.000 | 0.059 | 0.288 | 0.001 | 0.000 | 0.029 | 0.000 | 0.079 | 0.000 | 0.036 | 0.018 |
| HOP | 0.004 | 0.106 | 0.000 | 0.031 | 0.000 | 0.102 | 0.000 | 0.000 | 0.184 | 0.080 | 0.001 | 0.000 | 0.000 | 0.000 | 0.028 | 0.107 | 0.040 | 0.014 |
| IRO | 0.030 | 0.000 | 0.104 | 0.079 | 0.000 | 0.000 | 0.000 | 0.000 | 0.159 | 0.000 | 0.001 | 0.000 | 0.164 | 0.000 | 0.019 | 0.000 | 0.035 | 0.015 |
| IXL | 0.181 | 0.037 | 0.000 | 0.001 | 0.033 | 0.015 | 0.000 | 0.000 | 0.000 | 0.014 | 0.148 | 0.000 | 0.000 | 0.000 | 0.000 | 0.010 | 0.028 | 0.014 |
| KNO | 0.030 | 0.044 | 0.027 | 0.000 | 0.002 | 0.092 | 0.061 | 0.124 | 0.253 | 0.045 | 0.166 | 0.156 | 0.165 | 0.000 | 0.031 | 0.016 | 0.076 | 0.019 |
| LEA | 0.061 | 0.001 | 0.154 | 0.057 | 0.098 | 0.165 | 0.070 | 0.001 | 0.216 | 0.034 | 0.035 | 0.076 | 0.000 | 0.079 | 0.045 | 0.000 | 0.068 | 0.016 |
| LOI | 0.180 | 0.045 | 0.093 | 0.030 | 0.012 | 0.166 | 0.000 | 0.066 | 0.200 | 0.151 | 0.117 | 0.000 | 0.000 | 0.029 | 0.066 | 0.080 | 0.077 | 0.017 |
| NEW | 0.050 | 0.131 | 0.000 | 0.025 | 0.000 | 0.080 | 0.000 | 0.141 | 0.109 | 0.166 | 0.000 | 0.000 | 0.000 | 0.069 | 0.179 | 0.106 | 0.066 | 0.016 |
| NOR | 0.155 | 0.000 | 0.000 | 0.000 | 0.034 | 0.063 | 0.244 | 0.001 | 0.040 | 0.132 | 0.029 | 0.024 | 0.016 | 0.082 | 0.000 | 0.025 | 0.053 | 0.017 |
| NUL | 0.000 | 0.000 | 0.008 | 0.000 | 0.000 | 0.000 | 0.054 | 0.000 | 0.000 | 0.000 | 0.001 | 0.000 | 0.000 | 0.000 | 0.156 | 0.000 | 0.014 | 0.010 |
| OPH | 0.000 | 0.000 | 0.000 | 0.000 | 0.038 | 0.207 | 0.017 | 0.001 | 0.000 | 0.101 | 0.001 | 0.000 | 0.078 | 0.000 | 0.001 | 0.052 | 0.031 | 0.014 |
| PEB | 0.002 | 0.013 | 0.000 | 0.000 | 0.022 | 0.111 | 0.000 | 0.106 | 0.122 | 0.133 | 0.077 | 0.000 | 0.124 | 0.000 | 0.050 | 0.057 | 0.051 | 0.013 |
| RAG | 0.133 | 0.000 | 0.000 | 0.101 | 0.000 | 0.200 | 0.033 | 0.169 | 0.000 | 0.023 | 0.111 | 0.000 | 0.084 | 0.000 | 0.000 | 0.000 | 0.053 | 0.017 |
| SHA | 0.000 | 0.000 | 0.000 | 0.036 | 0.000 | 0.000 | 0.000 | 0.001 | 0.000 | 0.063 | 0.001 | 0.000 | 0.000 | 0.059 | 0.151 | 0.074 | 0.024 | 0.011 |
| SHE | 0.007 | 0.073 | 0.000 | 0.021 | 0.000 | 0.000 | 0.066 | 0.030 | 0.225 | 0.014 | 0.132 | 0.000 | 0.071 | 0.000 | 0.134 | 0.000 | 0.048 | 0.016 |
| TAP | 0.302 | 0.000 | 0.104 | 0.000 | 0.000 | 0.000 | 0.000 | 0.000 | 0.000 | 0.021 | 0.064 | 0.000 | 0.123 | 0.000 | 0.066 | 0.014 | 0.043 | 0.020 |
| *Acacia spondylophylla* | AS02 | AS23 | AS39 | AS51 | AS05 | AS09 | AS14 | AS24 | AS15 | AS20 | AS26 | AS34 | AS21 | AS28 | AS35 | AS42 | Mean | SE |
| BAT | 0.000 | 0.000 | 0.000 | 0.000 | 0.000 | 0.103 | 0.000 | 0.188 | 0.000 | 0.000 | 0.001 | 0.074 | 0.000 | 0.000 | 0.000 | 0.000 | 0.023 | 0.013 |
| DOO | 0.000 | 0.000 | 0.000 | 0.000 | 0.000 | 0.147 | 0.000 | 0.000 | 0.000 | 0.014 | 0.000 | 0.001 | 0.000 | 0.129 | 0.000 | 0.000 | 0.018 | 0.012 |
| DRI | 0.067 | 0.000 | 0.130 | 0.010 | 0.080 | 0.000 | 0.066 | 0.000 | 0.098 | 0.001 | 0.025 | 0.062 | 0.000 | 0.000 | 0.001 | 0.028 | 0.036 | 0.011 |
| KUN | 0.000 | 0.000 | 0.001 | 0.000 | 0.000 | 0.047 | 0.019 | 0.179 | 0.000 | 0.082 | 0.000 | 0.263 | 0.070 | 0.000 | 0.000 | 0.000 | 0.041 | 0.019 |
| LOI | 0.000 | 0.000 | 0.001 | 0.000 | 0.000 | 0.000 | 0.000 | 0.001 | 0.000 | 0.000 | 0.000 | 0.093 | 0.000 | 0.000 | 0.001 | 0.001 | 0.006 | 0.006 |
| MUL | 0.109 | 0.063 | 0.104 | 0.052 | 0.000 | 0.000 | 0.000 | 0.000 | 0.072 | 0.045 | 0.112 | 0.037 | 0.000 | 0.000 | 0.066 | 0.094 | 0.047 | 0.011 |
| MUN | 0.000 | 0.087 | 0.000 | 0.085 | 0.000 | 0.078 | 0.000 | 0.105 | 0.027 | 0.000 | 0.035 | 0.169 | 0.042 | 0.102 | 0.000 | 0.000 | 0.046 | 0.013 |
| NAM | 0.057 | 0.000 | 0.001 | 0.000 | 0.000 | 0.045 | 0.001 | 0.000 | 0.025 | 0.000 | 0.000 | 0.132 | 0.324 | 0.000 | 0.000 | 0.231 | 0.051 | 0.024 |
| NEW | 0.278 | 0.205 | 0.001 | 0.060 | 0.000 | 0.000 | 0.242 | 0.310 | 0.000 | 0.000 | 0.001 | 0.000 | 0.356 | 0.084 | 0.000 | 0.000 | 0.096 | 0.033 |
| NOR | 0.000 | 0.000 | 0.069 | 0.000 | 0.014 | 0.126 | 0.000 | 0.233 | 0.028 | 0.000 | 0.066 | 0.042 | 0.000 | 0.000 | 0.031 | 0.106 | 0.045 | 0.016 |
| NUL | 0.000 | 0.000 | 0.001 | 0.026 | 0.000 | 0.000 | 0.000 | 0.132 | 0.036 | 0.000 | 0.000 | 0.098 | 0.004 | 0.000 | 0.081 | 0.000 | 0.024 | 0.011 |
| PAN | 0.036 | 0.015 | 0.001 | 0.000 | 0.016 | 0.210 | 0.093 | 0.069 | 0.035 | 0.001 | 0.001 | 0.230 | 0.040 | 0.022 | 0.000 | 0.111 | 0.055 | 0.018 |
| SOL | 0.001 | 0.000 | 0.005 | 0.000 | 0.020 | 0.001 | 0.000 | 0.001 | 0.000 | 0.000 | 0.000 | 0.001 | 0.000 | 0.000 | 0.001 | 0.000 | 0.002 | 0.001 |
| WEE | 0.000 | 0.010 | 0.084 | 0.058 | 0.006 | 0.183 | 0.000 | 0.000 | 0.216 | 0.000 | 0.000 | 0.000 | 0.000 | 0.000 | 0.000 | 0.049 | 0.038 | 0.017 |
| WIT | 0.043 | 0.111 | 0.000 | 0.058 | 0.000 | 0.000 | 0.008 | 0.000 | 0.072 | 0.000 | 0.000 | 0.211 | 0.000 | 0.000 | 0.000 | 0.000 | 0.031 | 0.015 |
